# Supplementary material for: Record breaking achievements by spiders and the scientists who study them
Source: PeerJ. 2017 Oct 31;5:e3972. doi: 10.7717/peerj.3972 (PMC5668680; doi:10.7717/peerj.3972)
Supplement: Supplemental Information 1 — This file contains comments about the official biological records held by spiders as listed in the Guinness World Records database (www.guinnessworldrecords.com). For each record, we report the record holder according to the Guinness World Records database, as ofOctober 4th, 2017. We hereby list: –Official biological records confirmed by scientific literature, which we have included in the Spider World Records.–Official biological records that we consider incorrect, i.e. needing an update in the Guinness World Records. Marked with one asterisk (*).–Official biological records that we consider incomplete, e.g., for which we found new information or added specifications (see main text for details). Marked with two asterisks (**).–Official biological records that we have not reported due to low pertinence to our work or to the impossibility to confirm them using scientific literature. Marked with three asterisks (***). [file peerj-05-3972-s001.pdf]

SUPPLEMENTARY MATERIALS 1

Record breaking achievements by spiders and the scientists who study them

Stefano Mammola, Peter Michalik, Eileen Hebets & Marco Isaia

This file contains comments about the official biological records held by spiders as listed in the Guinness World Records database (www.guinnessworldrecords.com).

For each record, we report the record holder according to the Guinness World Records database, as of October 4th, 2017. We hereby list:

- Official biological records **confirmed** by scientific literature, which we have included in the Spider World Records.
- Official biological records that we consider **incorrect**, i.e. needing an update in the Guinness World Records. Marked with one asterisk (*).
- Official biological records that we consider **incomplete**, e.g., for which we found new information or added specifications (see main text for details). Marked with two asterisks (**).
- Official biological records that we have **not reported** due to low pertinence to our work or to the impossibility to confirm them using scientific literature. Marked with three asterisks (***).

Full bibliographic information for the references cited herein can be found in the reference section of the main text.

- Rarest spider **

Record holder (GWR, 2017): *Adelocosa anops*, a cave-dwelling spider inhabiting a few caves in the Hawaiian island of Kauai, covering an area of 10.5 km^2. Censuses for this species has never documented more than 30 individuals.

Comment: if we consider only the range occupied by the spider, there are other species which can compete for the record (see "*Rarest spiders*" in the main text). For instance, *Nothophantes horridus* is recorded exclusively from two abandoned limestone quarries near Plymouth (UK), covering overall a surface of ca. 0.1 km^2 (Cardoso & Hilton-Taylor, 2015). Besides, it is worth noting that population counts of cave-dwelling species are often unreliable: caves are connected with rock fissures and other habitats inaccessible to men, which often precludes correct population estimations (Mammola & Isaia, 2017).

- Smallest spider **

Record holder (GWR, 2017): *Patu marplesi* — body length of 0.3 mm.

Comment: according to the original description, the male of *Patu digua* is smaller than *P. marplesi* (*cfr.* Forster, 1959 and Forster & Platnick, 1977). See also "*Smallest female adult spiders"* in the main text.

- Noisiest spider **

Record holder (GWR, 2017): the Europeam buzzing spider *Anyphaena accentuata*, which produce a buzzing sound as par of its courtship behaviour.

Comment: there are other suitable candidates. See "*Loudest spider*" for more information.

- Fastest spider *

Record holder (GWR, 2017): *Tegenaria gigantea* (=*Tegenaria atrica*), reaching a maximum running speed of 1.9 km/h.

Comment: the Maroccan flic-flac spider *Cebrennus rechenbergi* is faster than *Tegenaria*

gigantea.

- Oldest spider **

Record holder (GWR, 2017): *Theraphosa blondi*

Comment: see "*Longest life span"* for more information.

- Largest spider

Record holder (GWR, 2017): a male of *Theraphosa blondi*, having a recorded leg-span of 28 cm.

Comment: see "*Largest living spiders*" for more informations.

- Strongest spider ***

Record holder (GWR, 2017): the Californian trap-door spider *Bothriocyrtum californicum*, resisting a force 38 times its own weight attempting to open its trap-door

Comment: we did not find published evidence in scientific literature about this record.

- Most spinnerets for a spider

Record holder (GWR, 2017): Mesothelae, having four pairs of spinnerets.

- Largest spider family

Record holder (GWR, 2017): Salticidae (> 5,000 species)

- Smallest spider web

Record holder (GWR, 2017): Symphytognathidae (web less than 10 mm in diameter).

- Earliest fossil spiders

Record holder (GWR, 2017): Mesothelae, which existed 300 Myr ago.

- Highest living spider

Record holder (GWR, 2017): *Europhrys* omnisuperstes, found at 6,700 m on Mount Everest

- Strongest spider web

Record holder (GWR, 2017): web of the Darwin's bark spider *Caerostris darwini*

- Most venomous spider **

Record holder (GWR, 2017): *Atrax robustus*

Comment: see main text ("*Most venomous to humans*") for some specifications about this record.

- Largest spider species **

Record holder (GWR, 2017): *Theraphosa blondi* (see also "

Comment: when considering legspan, *Heteropoda maxima* is the record holder (see "*Largest living spiders*").

- Largest spider eyes

Record holder (GWR, 2017): *Deinopis* spp.

- Largest spider eggs *

Record holder (GWR, 2017): Genus *Mygalomorphus*

Comment: this genus does not exist (see WSC, 2017).

- Smallest spider eggs ***

Record holder (GWR, 2017): *Oonops domesticus*

Comment: we were not able to find evidence in scientific literature supporting this record — quantitative data about egg size are extremely scattered and rare.

- First spider web in space

Record holder (GWR, 2017): webs and individuals of two *Araneus diadematus* (Arabella and Anita)

- Least venomous spiders **

Record holder (GWR, 2017): Uloboridae, lacking venom glands.

Comment: Holarcheidae should also be added, given that they lack poison gland openings in their fangs.

- Most bioluminescent spider

Record holder (GWR, 2017): *Cosmophasis umbratica*

- Simplest spider web ***

Record holder (GWR, 2017): *Miagrammopes, Mastophora, Cladomelea akermani*

Comment: part of these records were included under the label "*Most creative hunting strategy*".

- Earliest orb weaver spider

Record holder (GWR, 2017): *Cretaraneus vitalbae*

- Most sociable spider

Record holder (GWR, 2017): *Anelosimus eximus*

- Largest spider web outdoor ***

Record holder (GWR, 2017): a cobweb covering 4.5 hectares

Comment: we were not able to find evidence in scientific literature supporting this record

- Most maternal mother **

Record holder (GWR, 2017): *Theridion sisyphium*

Comment: there are other potential holders for this record — matriphagy has evolved in at least six spider families (Schneider, 1996). See *"Best mother".*

- Oldest spider silk

Record holder (GWR, 2017): silk in amber from 120 Myr ago

- Most aquatic spider

Record holder (GWR, 2017): the water spider *Argyroneta aquatica*

- Largest fossil spider

Record holder (GWR, 2017): *Mongolarachne jurassica*

- First herbivorous spider **

Record holder (GWR, 2017): *Bagheera kiplingi*

Comment: see "Strangest diet" for some specifications.

- Largest spider web indoors ***

Record holder (GWR, 2017): a spider's web of 5.08 m

- Largest orb-web spider

Record holder (GWR, 2017): *Nephila komaci*, having a leg-span of up to 12 cm

- Smallest families of spiders **

Record holder (GWR, 2017): Huttoniidae, Sinopimoidae, Trogloraptoridae , all including a single species

Comment: Sinopimoidae is not a valid family anymore. The only species belonging to this family (*Sinopimoa bicolor)* was transferred to Linyphiidae (WSC, 2017).

- Most widespread widow spider ***

Record holder (GWR, 2017): *Latrodectus geometricus*

- Oldest spider gossamer

Record holder (GWR, 2017): cobweb encase in amber of 140 Myr old

- Oldest spider in amber

Record holder (GWR, 2017): a species of Linyphiidae

- Oldest recorded victim attacked in spider web by web's spider

Record holder (GWR, 2017): the parasitic wasp *Cascoscelio incassus*

- Oldest spider web with trapped prey

Record holder (GWR, 2017): a piece of amber of 110 Myr ago

- Largest piece of natural spider silk ***

Record holder (GWR, 2017): a piece of cloth of 3.3 x 1.2 m made from natural spider silk

- Best adaptation to seawater by a spider species **

Record holder (GWR, 2017): *Desis marina*

Comment: see also main text ("*Longest time under water*") for some specifications.

- Largest continuous area of spider webs ***

Record holder (GWR, 2017): huge interwoven webs of *Stegodyphus* covering vegetation in a continuous silken mass for several kilometers

Comment: we have found no evidence in scientific literature to support this record.

- Largest item of clothing woven from spider silk

Record holder (GWR, 2017): a lady's cape

- Most eggs laid by a spider in a single batch *

Record holder (GWR, 2017): Genus *Mygalomorphus* (3,000 eggs)

Comment: this genus does not exist (see WSC, 2017).

- Fewest eggs laid by a spider in a single batch *

Record holder (GWR, 2017): *Oonops domesticus*, laying 2 eggs

Comment: some eggsacs of *Telema tenella* contain one egg only (Juberthie, 1985).
